# Homogeneous Population of the Brown Alga *Sargassum polycystum* in Southeast Asia: Possible Role of Recent Expansion and Asexual Propagation

**DOI:** 10.1371/journal.pone.0077662

**Published:** 2013-10-17

**Authors:** Sze Wai Chan, Chi Chiu Cheang, Anong Chirapart, Grevo Gerung, Chea Tharith, Put Ang

**Affiliations:** 1 Marine Science Laboratory, Chinese University of Hong Kong, N.T., Hong Kong SAR, China; 2 Algal Bioresources Research Center, Department of Fishery Biology, Faculty of Fisheries, Kasetsart University, Chatuchak, Bangkok, Thailand; 3 Faculty of Fisheries and Marine Science, Sam Ratulangi University, Manado, Indonesia; 4 Research and Development Institute (MFReDI), Fisheries Administration, Phnom Penh, Cambodia; Institute of Biochemistry and Biology, Germany

## Abstract

Southeast Asia has been known as one of the biodiversity hotspots in the world. Repeated glacial cycles during Pleistocene were believed to cause isolation of marine taxa in refugia, resulting in diversification among lineages. Recently, ocean current was also found to be another factor affecting gene flow by restricting larval dispersal in animals. Macroalgae are unique in having mode of reproduction that differs from that of animals. Our study on the phylogeographical pattern of the brown macroalga *Sargassum polycystum* using nuclear Internal Transcribed Spacer 2 (ITS2), plastidal RuBisCO spacer (Rub spacer) and mitochondrial cytochrome oxidase subunit-III (Cox3) as molecular markers revealed genetic homogeneity across 27 sites in Southeast Asia and western Pacific, in sharp contrast to that revealed from most animal studies. Our data suggested that *S. polycystum* persisted in single refugium during Pleistocene in a panmixia pattern. Expansion occurred more recently after the Last Glacial Maximum and recolonization of the newly flooded Sunda Shelf could have involved asexual propagation of the species. High dispersal ability through floating fronds carrying developing germlings may also contribute to the low genetic diversity of the species.

## Introduction

Southeast Asia is well known to be a hotspot for biodiversity. This high biodiversity is believed to have been contributed by its complex geological history. The repeated glacial cycles during Pleistocene caused fluctuation of sea level. This resulted in the formation of exposed land (e.g. Sunda Shelf) in relatively shallow sea that connected adjacent islands to form land bridges [1]. Pacific and Indian Oceans were separated and this separation was suggested to be the main event causing species differentiation between the two Oceans. Meanwhile, isolated basins such as the South China Sea, Sulu Sea, Celebes Sea and Flores Sea served as refugia for marine taxa. Their isolation led to the diversification of lineages among populations [2–4]. Recent oceanic currents, such as the Indonesian Thoughflow, is found to be restricting the larval dispersal of marine animals, such as the stomatopods, between Java Sea and Flores Sea [3]. The high genetic diversity in marine taxa in Southeast Asia is thus caused not only by past isolations, but also by restricted larval dispersal by recent oceanic conditions. 

Most of the marine phylogeographical studies in Southeast Asia, however, were on animals. Seaweeds, which exhibit a largely different dispersal mode than the animals, may provide new insights in understanding the phylogeographical processes in this region. Oceanic circulation has a profound effect on much of the population structure of marine animals as genetic connectively is highly associated with their pelagic larval duration. Unlike animals, seaweeds produce immobile germlings after fertilization and dispersal distance is expected to be much shorter. This may lead to higher genetic differentiation among seaweed populations. In North Atlantic, extensive phylogeographical studies have been carried out using multilocus molecular markers on brown algae [5,6]. Not much can be said of such studies in Southeast Asia except for a recent published research on the red alga *Gracilaria changii* in Peninsular Malaysia [7]. 

There are currently more than 300 species of *Sargassum* [8] reported from Southeast Asia. This makes *Sargassum* an ideal tool for biogeographical and phylogeographical studies because of its wide distribution range and high species diversity. The dispersal mode of the genus Sa*rgassum* is also unique among macroalgae. While they have short dispersal distance (<1m) of their germlings [9], they can be dispersed in the form of drifting fronds carrying with them developing propagules [10]. With the frequent isolation of many local basins in Southeast Asia in the geological past and the recent existence of complex oceanic circulation that is associated with monsoon and directional current flows (e.g. Indonesian Thoughflow), it is worth investigating the phylogeography of this region using this unique group of marine organisms. The target species in this study, *Sargassum polycystum* C. Agardh has a wide distribution range over the Indo-Pacific region, and a large distribution record in Southeast Asia [11,12]. This study has therefore the following objectives: (1) to investigate the genetic diversity and population structure of *S. polycystum*, with focus in Southeast Asia; and (2), to evaluate the potential mechanisms involved in bringing about any genetic structure observed. Three molecular markers were used to achieve these objectives, including the nuclear Internal Transcribed Spacer 2 (ITS2), plastidal RuBisCo spacer (Rub spacer) and mitochondrial cytochrome oxidase subunit-III (Cox3).

## Materials and Methods

### Sample collection and DNA extraction

**Figure 1:**
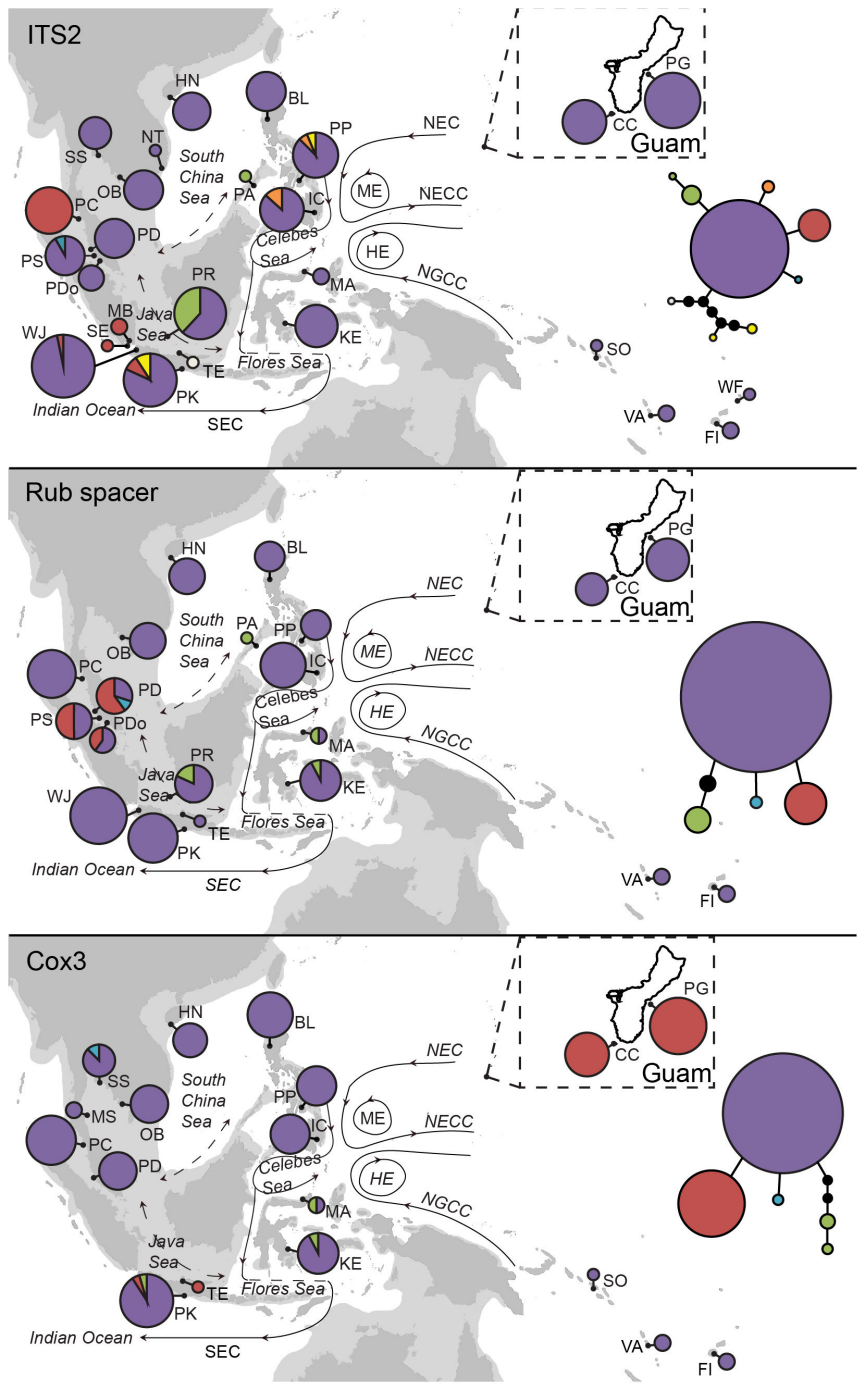
Haplotype distribution and haplotype network of *Sargassum* *polycystum* for ITS2, Rub spacer and Cox3. Pie chart size is proportional to sample size. Abbreviations for sample sites are given in Table S1 in File S1. Dominant currents are shown in solid lines [13] and seasonally reversing currents in dashed lines [14]. Light gray area shows the coastal outline during Pleistocene maximum low sea level of 120m [1]. SEC: South Equatorial Current, NEC: North Equatorial Current, NECC: North Equatorial Counter Current, NGCC: New Guinea Coastal Current, ME: Mindanao Eddy, HE: Halmahera Eddy.

Field studies did not involve any endangered or protected species. This study also involved collaborative works between institutions so no special permits were needed for sampling. Samples were collected from 22 sampling sites either by snorkeling or sampling during low tide (Figure 1; see also Table S1 in File S1). Individuals were collected more than 1m apart to avoid having the same mother plant as *Sargassum* germlings have short dispersal distance [9]. Leaf tips of 3-5 cm were dried and stored in silica gel for molecular analysis. The voucher plants were air-dried and deposited at the Simon F.S. Li Marine Science Laboratory Herbarium, The Chinese University of Hong Kong. Genomic DNA was extracted by modified cetyltrimethylammonium bromide (CTAB) method [2]. It was further purified by GENECLEAN II kit (Obiogene Inc.), following the manufacturer’s instructions.

### Polymerase Chain Reaction (PCR) and Sequencing

Primers for the three markers and PCR profiles were listed in [15]. All PCR products were sequenced in both directions with the same primers by Macrogen (Macrogen Inc., Seoul, Korea) employing the BigDye^TM^ terminator method. The presence of multiple peaks in chromatograms was checked in both directions to see whether there were intragenomic variations. Sequences were aligned and edited together with the sequences of *S. polycystum* available from GenBank by naked eyes using MEGA ver. 5 [16]. 

### Phylogeny and haplotype network construction

To ensure all the samples were *S. polycystum* with no misidentification before subsequent analysis, representative sequences from each haplotype of combined ITS2, Rub spacer and Cox3 sequences were initially screened to ensure they formed a monophyletic lineage and single taxonomic unit. The sequences were compared with those of other species in the subgenus *Sargassum* [15] using Bayesian Inference (BI) and Maximum Likelihood (ML) methods by MRBAYES 3.2.1 [17] and PhyML 3.0 [18] respectively. The Corrected Akaike information criterion (AICc) implemented in jMODELTEST 2.1.1 [19] was used to determine the best-fit substitution model. *Turbinaria ornata*, as suggested by Stiger and Horiguchi [20] and Phillips et al. [21], was used as outgroup. Any sequence that was not monophyletic was discarded to ensure consistency in subsequent analysis. 

To investigate the phylogenetic relationship within *S. polycystum*, Maximum Likelihood (ML) using PhyML 3.0 and Bayesian Inference (BI) using MRBAYES 3.2.1were applied. Best-fit substitution models were determined by jMODELTEST 2.1.1. *Sargassum ilicifolium* was used as outgroup as it is closely related within the same subgenus *Sargassum* [15]. Significance of the branching was evaluated by 1000 bootstrap in ML and posterior probability in BI analysis. For BI, two independent Markov Chain Monte Carlo (MCMC) searches were conducted until the divergence became small and stationary. Trees were sampled every 100 cycles in 1 500 000 generations and first 25% of the trees were discarded as burn-in. Haplotype networks for the three markers were generated to estimate gene genealogies using TCS 1.21 [22].

### Population structure analysis

Population structure of *S. polycystum* was analyzed by ARLEQUIN 3.5.1.2 [23]. Haplotype (h) and nucleotide (π) diversity [24,25] were calculated for each site. Tajima’s D [26] and Fu’s Fs [27] were calculated to test for the selective neutrality of the markers. Tajima’s D can distinguish between a recent population bottleneck and population expansion. Fu’s Fs is more sensitive to recent population expansion than Tajima’s D which gives large Fs negative values for excess number of alleles. Pairwise Φ_ST_ was calculated to determine the level of gene flow between populations. Hierarchical Analysis of Molecular Variance (AMOVA) with 10,000 permutations was conducted based on Φ_ST_ to find out the best spatial groupings of populations. Several groupings were tested iteratively, based on geographical proximity of the collecting sites, possible connectivity by current flow and haplotype similarity, to investigate whether there were any biogeographical barriers in between groups or localized haplotypes within groups that shaped the population structure. Each iteration was repeatedly tested by AMOVA until the highest percentages of variances between groups could be obtained. In addition, sampling sites were divided into smaller subregions corresponding to known marine basins or putative refugia, in order to investigate the direction and amount of gene flow between them. These subregions included South China Sea and Gulf of Thailand (BL, PP, PA, HN, NT, OB, SS, MS; see Figure 1 for site location), West Coast of Malay Peninsula (PC, PD, PS, PDo), West Java (WJ, SE, MB, PK, TE, PR), Celebes Sea and Flores Sea (IC, MA, KE), Guam (CC, PG) and Pacific Islands (SO, VA, FI, WF). All haplotypes in the populations were analyzed using the software MIGRATE 3.6 [28,29]. For each molecular marker, Bayesian search strategy was used in single 1,000,000 step chain with sampling increment of 100. Default setting was used, assuming unrestricted dispersal, to estimate number of immigrants per generation as calculated by multiplying θ (mutation-scaled effective population size) with M (mutation-scaled effective immigration rate) [28,29]. Samples of initial 25% were discarded as burn-in. Two independent runs were conducted and the results combined to represent >10,000 effective population size. 

A Mantel test [30] with 10,000 permutations was applied to test for the isolation by distance model by comparing the pairwise Φ_ST_ and the matrix of geographical distances among sampling sites. Geographical distances were measured using the shortest distance between two sites by Google Earth (http://www.google.com/earth/index.html). 

### Demographic history

Demographic history was only conducted for Cox3 data as there was no molecular clock available for ITS2 and Rub spacer. Mismatch distribution was used to detect any recent population expansion which is indicated with a unimodel distribution [31] using ARLEQUIN 3.5.1.2. Time of expansion (in generation), t, can be estimated by τ= 2μt. τ is the crest of mismatch distribution, μ is the mutation rate of the marker per generation. In addition, mismatch distribution was also conducted in ITS2 and Rub spacer without calculating the time of expansion. A Bayesian Skyline Plot of Cox3 was generated with BEAST 1.5.3 [32] using Markov Chain Monte Carlo (MCMC) sampling procedures with 10^8^ steps, sampled at every 1000 steps. Runs were repeated until effective sample size > 200 was reached in all parameters, as recommended in the user manual [33]. All runs were pooled in LogCombiner 1.7.3 with first 10% of generations discarded as burn-in. The Bayesian Skyline Plot was generated and visualized in Tracer 1.5.

The mutation rate of Cox3 of *Sargassum* was calculated based on the psbA clock (0.08%-0.12%/Myr) calibrated for *Fucus* [5]. PsbA sequences of *S. ilicifolium* were used as this species is closely related to *S. polycystum* within the same subgenus [15]. Divergence of psbA sequences between *S. ilicifolium* and *F. vesiculosus* (Genbank accession no: DQ307679) calculated was 5.67%. Therefore the estimated divergence time was 47.25 to 79.88 Myr (5.67/0.12 and 5.67/0.0.8 respectively). Divergence of Cox3 sequences between *S. ilicifolium* and *F. vesiculosus* (AY494079) was 14.7% and the estimated divergence rate of Cox3 was 0.207 to 0.311%/Myr (14.7/79.88 and 14.7/47.25 respectively). As the mutation rate should be half of the divergence rate, the estimated mutation rate was 1.035×10^-9^ to 1.555×10^-9^ substitutions per site per year. The generation time of 1 year was used as most *Sargassum* species have an annual cycle of growth and regeneration [34] and *S. polycystum* was shown to have a unimodal growth cycle [35]. 

## Results

### Genetic diversity

 A total of 261, 191 and 185 sequences were obtained from 27 sampling sites and Genbank for ITS2 (294 bp), Rub spacer (163 bp) and Cox3 (379 bp) respectively (Table S1 in File S1). Cox3 showed the highest genetic variability with highest haplotype and nucleotide diversity (H = 0.40 ± 0.04, π = 0.089 ± 0.082). Rub spacer had the lowest variability (H = 0.18 ± 0.04, π = 0.079 ± 0.101). There were 9, 4, 5 haplotypes for ITS2, Rub spacer and Cox3 respectively with 13, 3, 6 polymorphic sites. No intragenomic variations were observed for all the markers as all the chromatographs showed clear and consistent peaks in both sequencing directions.

### Interspecific and intraspecific phylogenetic relationship

HKY+G model determined by jMODELTEST was used to analyze the taxonomic status of haplotypes from combined sequences of ITS2, Rub spacer and Cox3. Phylogenetic trees inferred from Bayesian Inference and Maximum Likelihood methods with other species in the subgenus *Sargassum* showed that sequences from this study were monophyletic (see Figure S1). All sequences thus were used for subsequent analysis. HKY, F81+I and HKY models were found to be optimum for ITS2, Rub spacer and Cox3 respectively. ML and BI analyses consistently gave same tree topology for all markers with a single clade revealed (see Figures S2-S4).

### Population structure

**Figure 2:**
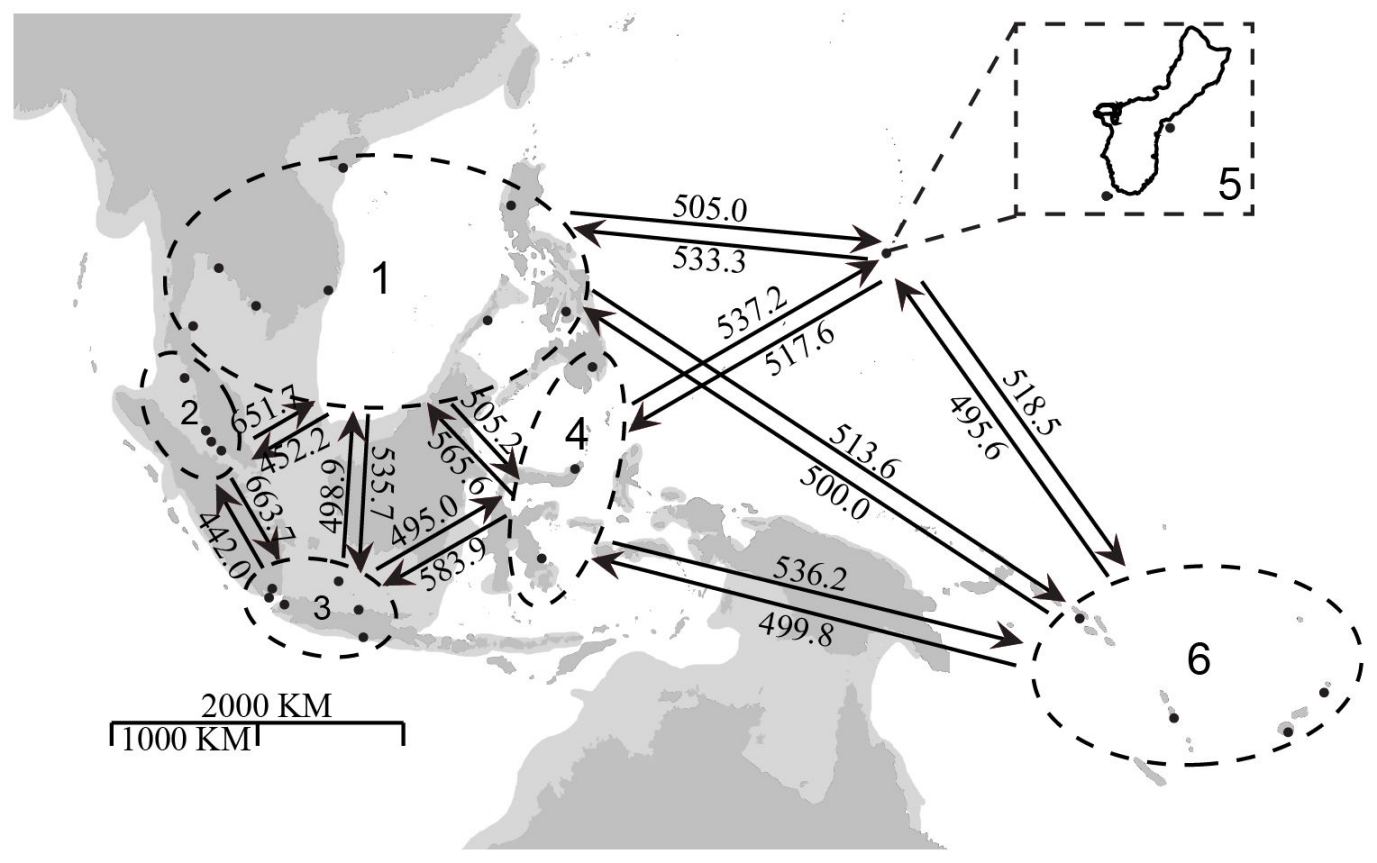
Subregions of the study area defined for MIGRATE analysis. 1: South China Sea and Gulf of Thailand; 2: West Coast of Malay Peninsula; 3: West Java; 4: Celebes Sea and Flores Sea; 5: Guam; 6: Pacific Islands. Directionality of gene flows between selected subregions based on Cox3 is shown with N_e_, the mean number of effective migrants per generation. All values are within the range of 440 to 665. Other details of gene flow among subregions based on all three markers are listed in Tables S6-S8 in File S1 respectively.

 Tajima’s D and Fu’s Fs gave significant negative value for ITS2 and insignificant negative value for Rub spacer and Cox3 (Table S1 in File S1). Neutrality tests indicated there may have been recent population expansion for *S. polycystum*. Most of the populations showed non-significant pairwise Φ_ST_ values in all markers indicating shallow population structure (Tables S2-S4 in File S1). Result of hierarchical AMOVA is summarized in Table S5 in File S1. No groupings give significant population structure in ITS2 and Rub spacer. For Cox3, highest percentage of variance (85.38%) was found with the groupings: (1) Southeast Asia; (2) Guam; (3) Pacific Islands. MIGRATE analysis for all markers showed comparable rates of import and export of migrants between subregions (Figure 2 , Tables S6-8 in File S1), indicating a balanced gene flow among these subregions. Mantel test for ITS2, Rub spacer and Cox3 showed low regression coefficient (r = -3.8 × 10^-5^; r = -2.7× 10^-5^, r = -1.9 × 10^-5^, p > 0.05 respectively) that did not fit the isolation by distance model.

### Demographic history

**Figure 3:**
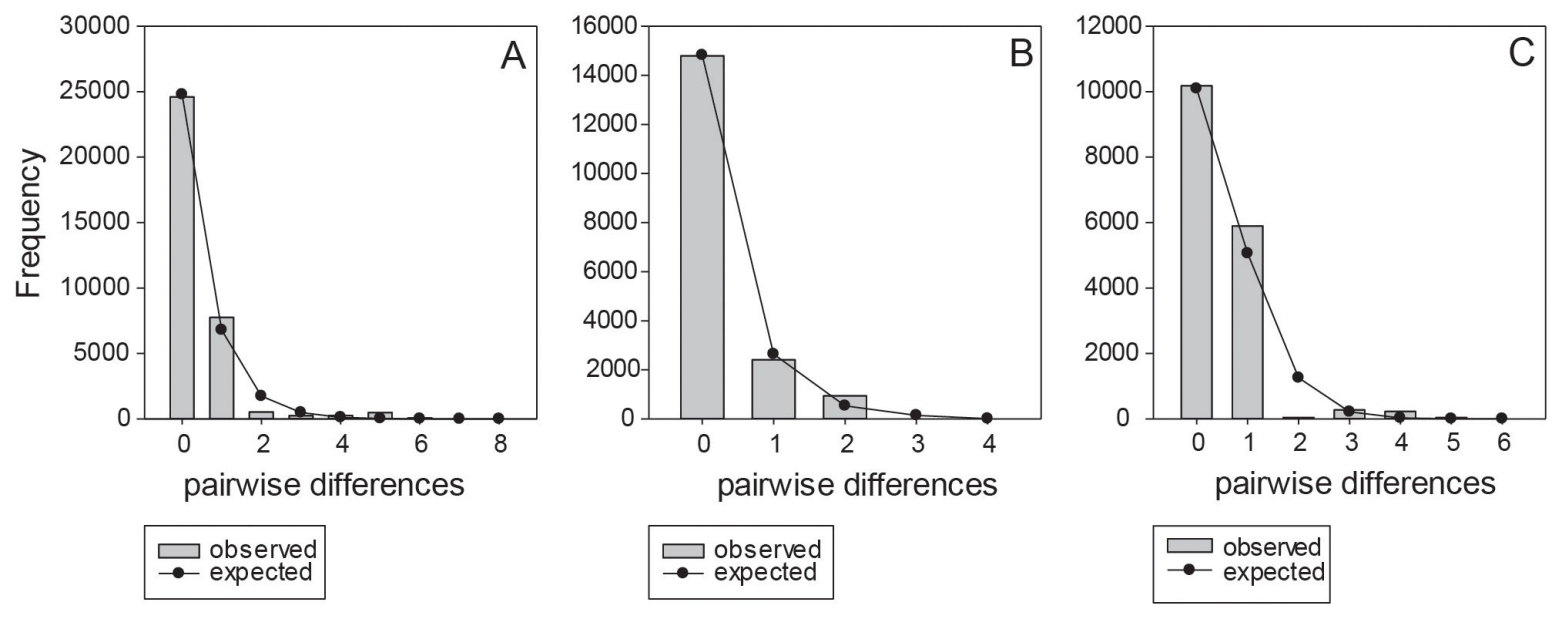
Mismatch distribution of *Sargassum* *polycystum* using ITS2, Rub spacer and Cox3. Bar chart and line graph indicate the observed and expected frequency respectively.

Mismatch distribution of all markers fit the sudden expansion model (Figure 3, ITS2: Sum of squared deviation = 0.00260, p>0.05; Rub spacer: Sum of squared deviation = 0.000894, p>0.05; Cox3: Sum of squared deviation = 0.00814, p>0.05). Estimated expansion time calculated from Cox3 was 0.62- 0.41Mya, middle Pleistocene (τ = 0.484, mutation rate = 1.035×10^-9^ to 1.555×10^-9^ substitutions per site per year, length of Cox3 sequence =379). Bayesian Skyline Plot did not show any recent population expansion reflected by a flat curve (Figure S5). 

## Discussion

### Genetic variability of S. *polycystum*

The overall genetic diversity of *S. polycystum* is relatively low compared to that of other brown macroalgae. For examples, 51 haplotypes of cox3 were revealed in *Sargassum horneri* [36], 17 were found in *Ishige okamurae* [37] and nine were found in *Undaria pinnatifida* [38] in Northwestern Pacific. In Southeast Asia, study on *Gracilaria changii* using Cox1 gene revealed six haplotypes in Peninsular Malaysia [7]. The low genetic diversity of *S. polycystum* is unexpected as the sampling area in the present study is much larger than those in previous studies. Study on *S. ilicifolium*, having similar sampling sites as the present study, revealed 16, 8, 17 haplotypes for ITS2, Rub spacer and Cox3 respectively (Chan et al., unpublished data). This indicated that the molecular markers used in this study were variable enough to give significant population structures in other closely related *Sargassum* species and homogeneity found in *S. polycystum* is not an artifact of the markers used. The difference in genetic diversity may reflect a difference in evolutionary history experienced by *S. polycystum* and/or the physical environment it is subjected to. 

 Among the three molecular markers used, Rub spacer showed lowest genetic variability that is comparable to that in other studies on *Sargassum* [2,36,39]. This showed that Rub spacer has low mutation rate and may not be suitable for phylogeographical studies of *Sargassum*. On the other hand, genetic variabilities of ITS2 and Cox3 found in the present study are similar (H = 0.28 ± 0.04, π = 0.030 ± 0.032; H = 0.39 ± 0.03, π =0.079 ± 0.076). 

### Population Structure

Overall, the population structure of *S. polycystum* is relatively homogeneous with few haplotypes derived from the main centre haplotype (Figure 1). In contrast, for animals, high genetic diversity is observed in giant clam [40,41], stomatopods [3], clownfish [4] and coral [42] within the same region due to past isolation in refugia and recent oceanographic currents which limited gene flow. For example, exposed Sunda Shelf during Last Glacial Maximum caused isolation of clownfish populations between Sulawesi and Indo-Malay Archipelago [4] and downward directional flow of Indonesian Throughflow maintained the genetic connectivity of stomatopod populations along the west coast of Sulawesi and East Java [3]. Phylogeographical analysis of three *Sargassum* spp. in northwestern Pacific [2,36,43] also showed *Sargassum* populations could both be structured by Pleistocene isolation and postglacial recolonization associated with current flows (e.g. Kuroshio Current). These factors, however, seem to have little effect on *S. polycystum* as no significant population structure can be observed within the Southeast Asian region. No population isolation is observed in Sulawesi and other identified refugia (e.g. South China Sea and Sulu Sea) and the Indonesian Throughflow did not appear to impose strong restricted dispersal between Java Sea and Flores Sea. This lack of population structure is further supported by both AMOVA and MIGRATE results. AMOVA did not identify clear groupings of *S. polycyctum* populations within Southeast Asian region so AMOVA results could not be used as a reference to estimate the gene flows between groups. Nonetheless, subregions based on putative marine basins or refugia were used in MIGRATE analysis and the results also revealed relatively balanced gene flows between subregions. 

Different from the marine animals studied, *S. polycystum* is capable of both sexual and asexual reproductions. It was reported that *S. polycystum* can reproduce asexually with new main axes growing out from its ramifying holdfast (stolons) [44]. Indeed, our own experiments also indicated that fragments of this ramifying holdfast (stolons) can develop new shoots and new axes, which subsequently could reattach to the substrata (Ang et al, unpublished data). This feature is unique and uncommon in the genus *Sargassum* [11]. Interestingly, *S. hemiphyllum* var. *chinensis*, which also has a ramifying holdfast, also showed a low genetic diversity within its range of distribution in southern China [2]. Asexual propagation of *S. polycystum* could thus be suggested to be involved in maintaining the genetic homogeneity of the species. Similar process has also been suggested for the brown alga *Fucus vesiculosus* [45]. However, the ratio of sexual and asexual reproductions in the populations of *S. polycystum* remains unknown. Calculation of incidence of asexual vs. sexual dispersal is difficult as comparison with known asexually vs. sexually propagated populations of other *Sargassum* species within the same distribution range is needed. To date, however, only *S. polycystum* is putatively known to propagate asexually. Further investigation using multilocus genetic markers would also be needed to further confirm the roles of asexual propagation.

Asexual propagation alone would not be sufficient to explain the lack of genetic structure in *S. polycystum* populations across such a big geographical region covered in the present study. Homogeneity of the populations could also indicate high dispersal ability of the species as no isolation by distance was observed in *S. polycystum* nor was there distinct directional gene flow revealed by MIGRATE analysis. For animal species, population structures are determined by larval duration and current flow. In contrast, *Sargassum* is capable of dispersing for long distance in the form of drifting fronds with germlings [10] and rapid recolonzation of a site could be facilitated. Homogeneity was also observed in other rafting seaweeds. For example, samples from subantarctic islands of Southern bull kelp *Durvillaea antarctica* showed homogeneity dominated by one single haplotype over a distance of 10,000 km [46]. High genetic connectivity was also observed in the giant kelp *Macrocystis pyrifera* along the subantarctic islands and southern-central Chile [47] with high degree of shared haplotypes. All these can be explained by high dispersal ability of kelp rafts [47]. In northwestern Pacific, drifting of *Sargassum horneri* with coastal currents was found to contribute genetic connectivity in East China Sea and allowed secondary contacts between the Chinese and Japanese populations [36]. In contrast, study in central Philippines revealed 21 cryptic species of the red alga *Portieria*, suggesting that speciation may occur within < 100 km due to the absence of propagules, which in turn limits the dispersal ability of the populations [48]. High endemism was thus observed with no or limited overlaps of species even though islands in central Philippines are highly connected by ocean currents. The dispersal mode of *Sargassum*, having drifting fronds with germlings, suggests that it has a much higher dispersal potential than *Portieria*. The long persistence of drifting fronds of *Sargassum* allowed eventual mixing of isolated populations (e.g. by Indonesian Throughflow) and explains the homogeneity across long distance, i.e. from Pacific Islands to Southeast Asia.

Nonetheless, despite an overall homogeneous pattern of *S. polycystum* populations observed, small discrepancies could still be found in the Philippine, Indonesian and Thai populations based on ITS2 and Guam and eastern Indonesian populations based on Cox3. These discrepancies were not consistent between the patterns generated from ITS2 and Rub spacer and those from Cox3 (Figure 1). In either situation, some isolated cases of mutation had occurred in these populations but their dispersal was restricted. For example, there appears to have a single base pair mutation in the Guam population, as suggested by Cox3. By and large, the Guam population could have been restricted in its dispersal by the directional flow of the North Equatorial Current (NEC in Figure 1). However, some individuals could have gotten into the Indonesia Throughflow and eventually reached Java (TF and PK). The frequency of the Guam haplotype in Javan populations is very low and the absence of this haplotype in any sites between Guam and Java may simply be a sampling problem. 

### Demographic history

Star-shaped haplotype network (Figure 1) and mismatch distribution of Cox3 (Figure 3) indicated demographic expansion of *S. polycystum* populations in mid-Pleistocene (0.62- 0.41Mya) that remained constant recently, as inferred from Bayesian Skyline Plot (Figure S5). The homogeneous pattern resembles null model I of panmixia proposed by Maggs et al. [49]. This model suggested no significant population differentiation but with only shallow mixture of derived haplotypes. Panmixia was also found in the European eel, *Anguilla anguilla* L. with low genetic differentiation between populations (FST = 0.0014; p<0.01) [50]. It can be suggested that population of *S. polycystum* retracted during mid-Pleistocene and persisted in a single refugium which would have included the Philippines and western Pacific islands. After the Last Glacial Maximum, recolonization of the rest of the slowly flooded Sunda Shelf was likely achieved mainly through asexual propagation and rapid dispersal in the form of drifting fronds with germlings. Mutation of the species was slow resulting in homogeneity of the populations across the whole Southeast Asian region. 

## Conclusion

The absence of significant population structure in *S. polycystum* over a wide area in Southeast Asia and western Pacific suggests high genetic connectivity within this region. Asexual propagation and high dispersal ability are proposed to be the mechanisms that contributed to this observed phenomenon. There appears to be no significant isolation of *S. polycystum* populations during the lowering of sea levels and demographic expansion may have occurred more recently, after the Last Glacial Maximum. The species could have persisted in a single refugium before recolonization occurred. To further reveal the evolutionary history of this species in Southeast Asia, physiological experiment on the ratio of sexual and asexual reproduction would be needed. Multilocus molecular markers with higher variability (e.g. microsatellites) are suggested to reveal the hidden diversity among the populations. More sampling sites, especially in Indian Ocean, may also help to elucidate the extent of population homogeneity in this species.

## Supporting Information

File S1 **Combined file containing all supporting tables.** Table S1: Sample localities, diversity indices and neutrality tests of Sargassum *polycystum* using ITS2, Rub spacer and Cox3 (from top to bottom) including sequences from Genbank. Table S2: Pairwise Φ_ST_ among populations of Sargassum *polycystum* based on ITS2. Table S3: Pairwise Φ_ST_ among populations of Sargassum *polycystum* based on Rub spacer. Table S4: Pairwise Φ_ST_ among populations of Sargassum *polycystum* based on Cox3. Table S5: Hierarchical Analysis of Molecular Variance (AMOVA) based on the three markers ITS2, Rub spacer and Cox3 from Sargassum *polycystum*. Table S6: Migration estimates of gene flow between subregions 1 to 6 and 95% confidence values as estimated by MIGRATE using ITS2. Table S7: Migration estimates of gene flow between subregions 1 to 6 and 95% confidence values as estimated by MIGRATE using Rub spacer. Table S8: Migration estimates of gene flow between subregions 1 to 6 and 95% confidence values as estimated by MIGRATE using Cox3.
(DOCX)

Figure S1 **Phylogenetic tree of representative sequences from each haplotype of combined ITS2, Rub spacer and Cox3 sequences from *Sargassumpolycystum* with other *Sargassum* spp.** **in subgenus *Sargassum***.
Voucher numbers of sequences from Genbank are presented after species name. Representative sequences from each haplotype of *S. polycystum* are labeled in black bar. *Turbinaria ornata* is used as outgroup. Posterior probabilities of Bayesian Inference and boostrap value of Maximum Likelihood are shown. Tables of sample localities, diversity indices, neutrality tests; Pairwise <PHI>ST; Hierarchical Analysis of Molecular Variance (AMOVA) and Migration estimates of gene flow between subregions of three genes of *S. polycystum*.
(TIF)

Figure S2 **Phylogenetic tree inferred from ITS2 using *Sargassumilicifolium* as outgroup.** Posterior probabilities of Bayesian Inference and boostrap values of Maximum likelihood are shown.
(TIF)

Figure S3 **Phylogenetic tree inferred from Rub spacer using *Sargassumilicifolium* as outgroup.** Posterior probabilities of Bayesian Inference and boostrap values of Maximum likelihood are shown.
(TIF)

Figure S4 **Phylogenetic tree inferred from Cox3 using *Sargassumilicifolium* as outgroup.** Posterior probabilities of Bayesian Inference and boostrap values of Maximum likelihood are shown.
(TIF)

Figure S5 **Bayesian Skyline Plot of Cox3 in effective population size with function of time (year before present).** 95% confidence interval is shown in blue.
(TIF)
