# Supplementary material for: Homogeneous Population of the Brown Alga Sargassum polycystum in Southeast Asia: Possible Role of Recent Expansion and Asexual Propagation
Source: PLoS One. 2013 Oct 17;8(10):e77662. doi: 10.1371/journal.pone.0077662 (PMC3798308; doi:10.1371/journal.pone.0077662)
Supplement: File S1 — Combined file containing all supporting tables. Table S1: Sample localities, diversity indices and neutrality tests of Sargassum polycystum using ITS2, Rub spacer and Cox3 (from top to bottom) including sequences from Genbank. Table S2: Pairwise ΦST among populations of Sargassum polycystum based on ITS2. Table S3: Pairwise ΦST among populations of Sargassum polycystum based on Rub spacer. Table S4: Pairwise ΦST among populations of Sargassum polycystum based on Cox3. Table S5: Hierarchical Analysis of Molecular Variance (AMOVA) based on the three markers ITS2, Rub spacer and Cox3 from Sargassum polycystum. Table S6: Migration estimates of gene flow between subregions 1 to 6 and 95% confidence values as estimated by MIGRATE using ITS2. Table S7: Migration estimates of gene flow between subregions 1 to 6 and 95% confidence values as estimated by MIGRATE using Rub spacer. Table S8: Migration estimates of gene flow between subregions 1 to 6 and 95% confidence values as estimated by MIGRATE using Cox3. (DOCX) [file pone.0077662.s001.docx]

**Table S1** Sample localities, diversity indices and neutrality tests of *Sargassum polycystum* using ITS2, Rub spacer and Cox3 (from top to bottom) including sequences from Genbank.

| **Locality** | **N*** | **H ± SD*** | **π± SD*** | ***D**** | ***Fs**** |
| --- | --- | --- | --- | --- | --- |
| 1. Bolinao, Philippines (BL) | 12 | 0.00 ± 0.00 | 0.000 ± 0.000 | 0.00 | N. A. |
|  | 7 | 0.00 ± 0.00 | 0.000 ± 0.000 | 0.00 | N. A. |
|  | 16 | 0.00 ± 0.00 | 0.000 ± 0.000 | 0.00 | N. A. |
| 1. Maribago, Cebu, Philippines (PP) | 16 | 0.24 ± 0.14 | 0.048 ± 0.045 | **-1.93** | 0.04 |
|  | 7 | 0.00 ± 0.00 | 0.000 ± 0.000 | 0.00 | N. A. |
|  | 12 | 0.00 ± 0.00 | 0.000 ± 0.000 | 0.00 | N. A. |
| 1. Puerto Princesa, Palawan, Philippines (PA) | 1 | 1.00 ± 0.00 | 0.000 ± 0.000 | 0.00 | N. A. |
|  | 1 | 1.00 ± 0.00 | 0.000 ± 0.000 | 0.00 | N. A. |
|  | 0 |  |  |  |  |
| 1. Indian's Cove, Talikud Island, Davao, Philippines (IC) | 15 | 0.25 ± 0.13 | 0.019 ± 0.026 | -0.40 | 0.13 |
|  | 16 | 0.00 ± 0.00 | 0.000 ± 0.000 | 0.00 | N. A. |
|  | 12 | 0.00 ± 0.00 | 0.000 ± 0.000 | 0.00 | N. A. |
| 1. Sanya, Hainan, China (HN) | 11 | 0.00 ± 0.00 | 0.000 ± 0.000 | 0.00 | N. A. |
|  | 10 | 0.00 ± 0.00 | 0.000 ± 0.000 | 0.00 | N. A. |
|  | 9 | 0.00 ± 0.00 | 0.000 ± 0.000 | 0.00 | N. A. |
| 1. Samaesan Sattahip Chon Buri, Thailand (SS) | 8 | 0.00 ± 0.00 | 0.000 ± 0.000 | 0.00 | N. A. |
|  | 0 |  |  |  |  |
|  | 8 | 0.25 ± 0.18 | 0.042 ± 0.059 | -1.05 | -0.18 |
| 1. Surat Thani Mud Sum Island, Thailand (MS) | 0 |  |  |  |  |
|  | 0 |  |  |  |  |
|  | 2 | 0.00 ± 0.00 | 0.000 ± 0.000 | 0.00 | N. A. |
| 1. Otres Beach, Sihanoukville, Cambodia (OB) | 12 | 0.00 ± 0.00 | 0.000 ± 0.000 | 0.00 | N. A. |
|  | 10 | 0.00 ± 0.00 | 0.000 ± 0.000 | 0.00 | N. A. |
|  | 11 | 0.00 ± 0.00 | 0.000 ± 0.000 | 0.00 | N. A. |
| 1. Cape Rachado, Port Dickson, Malaysia (PD) | 12 | 0.00 ± 0.00 | 0.000 ± 0.000 | 0.00 | N. A. |
|  | 10 | 0.60 ± 0.13 | 0.244 ± 0.223 | 0.12 | -0.10 |
|  | 11 | 0.00 ± 0.00 | 0.000 ± 0.000 | 0.00 | N. A. |
| 1. Pulau Dodol, Melaka, Malaysia (PDo) | 5 | 0.00 ± 0.00 | 0.000 ± 0.000 | 0.00 | N. A. |
|  | 5 | 0.60 ± 0.18 | 0.200 ± 0.220 | 1.22 | 0.63 |
|  | 0 |  |  |  |  |
| 1. Pulau Serimbun, Melaka, Malaysia (PS) | 12 | 0.17 ± 0.13 | 0.013 ± 0.021 | -1.14 | -0.48 |
|  | 10 | 0.56 ± 0.07 | 0.185 ± 0.186 | 1.46 | 1.10 |
|  | 0 |  |  |  |  |
| 1. Pantai Cenang, Langkawi, Malaysia (PC) | 17 | 0.00 ± 0.00 | 0.000 ± 0.000 | 0.00 | N. A. |
|  | 18 | 0.00 ± 0.00 | 0.000 ± 0.000 | 0.00 | N. A. |
|  | 19 | 0.00 ± 0.00 | 0.000 ± 0.000 | 0.00 | N. A. |
| 1. Merak Balantung, Indonesia (MB) | 2 | 0.00 ± 0.00 | 0.000 ± 0.000 | 0.00 | N. A. |
|  | 0 |  |  |  |  |
|  | 0 |  |  |  |  |
| 1. Teluk Awur, Indonesia (TE) | 1 | 1.00 ± 0.00 | 0.000 ± 0.000 | 0.00 | N. A. |
|  | 1 | 1.00 ± 0.00 | 0.000 ± 0.000 | 0.00 | N. A. |
|  | 1 | 1.00 ± 0.00 | 0.000 ± 0.000 | 0.00 | N. A. |
| 1. Sebuku, Indonesia (SE) | 1 | 1.00 ± 0.00 | 0.000 ± 0.000 | 0.00 | N. A. |
|  | 0 |  |  |  |  |
|  | 0 |  |  |  |  |
| 1. Merak, West Java, Indonesia (WJ) | 31 | 0.06 ± 0.06 | 0.005 ± 0.012 | -1.14 | -1.24 |
|  | 25 | 0.00 ± 0.00 | 0.000 ± 0.000 | 0.00 | N. A. |
|  | 0 |  |  |  |  |
| 1. Palau Pramuka, Indonesia (PR) | 21 | 0.53 ± 0.08 | 0.045 ± 0.042 | 0.14 | 0.14 |
|  | 11 | 0.33 ± 0.15 | 0.218 ± 0.205 | -0.13 | 1.45 |
|  | 0 |  |  |  |  |
| 1. Pantai Kukup, Indonesia (PK) | 22 | 0.33 ± 0.12 | 0.080 ± 0.062 | -1.14 | 1.41 |
|  | 19 | 0.00 ± 0.00 | 0.000 ± 0.000 | 0.00 | N. A. |
|  | 22 | 0.18 ± 0.11 | 0.076 ± 0.078 | **-1.99** | -0.33 |
| 1. Kendari, Sulawesi, Indonesia (KE) | 14 | 0.00 ± 0.00 | 0.000 ± 0.000 | 0.00 | N. A. |
|  | 13 | 0.15 ± 0.13 | 0.103 ± 0.127 | -1.47 | 0.36 |
|  | 13 | 0.15 ± 0.13 | 0.077 ± 0.081 | **-1.65** | 0.98 |
| 1. Manado, Sulawesi, Indonesia (MA) | 2 | 0.00 ± 0.00 | 0.000 ± 0.000 | 0.00 | N. A. |
|  | 2 | 1.00 ± 0.50 | 0.667 ± 0.816 | 0.00 | 0.69 |
|  | 2 | 1.00 ± 0.50 | 0.500 ± 0.577 | 0.00 | 1.10 |
| 1. Cocos Island, Guam (CC) | 15 | 0.00 ± 0.00 | 0.000 ± 0.000 | 0.00 | N. A. |
|  | 8 | 0.00 ± 0.00 | 0.000 ± 0.000 | 0.00 | N. A. |
|  | 15 | 0.00 ± 0.00 | 0.000 ± 0.000 | 0.00 | N. A. |
| 1. Pago Bay, Guam (PG) | 24 | 0.00 ± 0.00 | 0.000 ± 0.000 | 0.00 | N. A. |
|  | 14 | 0.00 ± 0.00 | 0.000 ± 0.000 | 0.00 | N. A. |
|  | 25 | 0.00 ± 0.00 | 0.000 ± 0.000 | 0.00 | N. A. |
| Genbank | N | N | π± SD | *D* | *Fs* |
| **23.** Fiji (FI) |  |  |  |  |  |
| ITS2 : EU833421-2 | 2 | 0.00 ± 0.00 | 0.000 ± 0.000 | 0.00 | N. A. |
| Rub : EU833471-2 | 2 | 0.00 ± 0.00 | 0.000 ± 0.000 | 0.00 | N. A. |
| Cox3 : EU833405, EU833410 | 2 | 0.00 ± 0.00 | 0.000 ± 0.000 | 0.00 | N.A. |
| **24.** Cau Da, Nhatrang, Vietnam (NT) |  |  |  |  |  |
| ITS2: AB043114 | 1 | 1.00 ± 0.00 | 0.000 ± 0.000 | 0.00 | N. A. |
| **25.** Solomon Islands (SO) |  |  |  |  |  |
| ITS2 : EU833423 | 1 | 1.00 ± 0.00 | 0.000 ± 0.000 | 0.00 | N. A. |
| Cox3 : EU833399 | 1 | 1.00 ± 0.00 | 0.000 ± 0.000 | 0.00 | N. A. |
| **26.**  Vanuatu (VA) |  |  |  |  |  |
| ITS2 : EU833419-20 | 2 | 0.00 ± 0.00 | 0.000 ± 0.000 | 0.00 | N. A. |
| Rub : EU833474-5 | 2 | 0.00 ± 0.00 | 0.000 ± 0.000 | 0.00 | N. A. |
| Cox3 : EU833387-8 | 2 | 0.00 ± 0.00 | 0.000 ± 0.000 | 0.00 | N. A. |
| **27.**  Wallis and Futuna (WF) |  |  |  |  |  |
| ITS2 : EU833418 | 1 | 1.00 ± 0.00 | 0.000 ± 0.000 | 0.00 | N.A. |
| Whole data set | 261  191  183 | 0.28 ± 0.04  0.18 ± 0.04  0.39 ± 0.03 | 0.030 ± 0.032  0.079 ± 0.101  0.079 ± 0.076 | **-1.95**  -0.87  -1.11 | **-6.61**  -1.83  -1.21 |

*Number of individuals (N), haplotype (h) and nucleotide (π) diversities, Tajima’s *D*, Fu’s *Fs* are given per site. Significant values (p < 0.05) are in bold.

**Table S2.** Pairwise Φ_ST_ among populations of *Sargassum polycystum* based on ITS2.

| Group* | Site | BL | PP | HN | PA | NT | IC | PR | TE | MA | KE | WJ | PK | SE | MB | PD | PDo | PS | PC | OB | SS | SO | VA | FI | WF | PG |
| --- | --- | --- | --- | --- | --- | --- | --- | --- | --- | --- | --- | --- | --- | --- | --- | --- | --- | --- | --- | --- | --- | --- | --- | --- | --- | --- |
| 1 | BL |  |  |  |  |  |  |  |  |  |  |  |  |  |  |  |  |  |  |  |  |  |  |  |  |  |
| 1 | PP | 0.000 |  |  |  |  |  |  |  |  |  |  |  |  |  |  |  |  |  |  |  |  |  |  |  |  |
| 1 | HN | 0.000 | 0.000 |  |  |  |  |  |  |  |  |  |  |  |  |  |  |  |  |  |  |  |  |  |  |  |
| 1 | PA | 1.000 | 0.524 | **1.000** |  |  |  |  |  |  |  |  |  |  |  |  |  |  |  |  |  |  |  |  |  |  |
| 1 | NT | 0.000 | 0.000 | 0.000 | 1.000 |  |  |  |  |  |  |  |  |  |  |  |  |  |  |  |  |  |  |  |  |  |
| 2 | IC | 0.051 | 0.000 | **0.042** | 0.782 | 0.000 |  |  |  |  |  |  |  |  |  |  |  |  |  |  |  |  |  |  |  |  |
| 2 | PR | **0.243** | **0.181** | **0.234** | 0.114 | 0.000 | **0.237** |  |  |  |  |  |  |  |  |  |  |  |  |  |  |  |  |  |  |  |
| 2 | TE | 1.000 | 0.804 | 1.000 | 1.000 | 1.000 | 0.921 | 0.828 |  |  |  |  |  |  |  |  |  |  |  |  |  |  |  |  |  |  |
| 2 | MA | 0.000 | 0.000 | 0.000 | 1.000 | 0.000 | 0.000 | 0.000 | 1.000 |  |  |  |  |  |  |  |  |  |  |  |  |  |  |  |  |  |
| 2 | KE | 0.000 | 0.000 | 0.000 | 1.000 | 0.000 | 0.065 | **0.261** | 1.000 | 0.000 |  |  |  |  |  |  |  |  |  |  |  |  |  |  |  |  |
| 3 | WJ | 0.000 | 0.033 | 0.000 | 0.938 | 0.000 | 0.094 | **0.333** | **0.979** | 0.000 | 0.000 |  |  |  |  |  |  |  |  |  |  |  |  |  |  |  |
| 3 | PK | 0.008 | 0.000 | 0.002 | 0.328 | 0.000 | 0.036 | **0.162** | 0.691 | 0.000 | 0.018 | 0.054 |  |  |  |  |  |  |  |  |  |  |  |  |  |  |
| 3 | SE | 1.000 | 0.524 | 1.000 | 1.000 | 1.000 | 0.782 | 0.587 | 1.000 | 1.000 | 1.000 | 0.933 | 0.238 |  |  |  |  |  |  |  |  |  |  |  |  |  |
| 3 | MB | **1.000** | 0.593 | 1.000 | 1.000 | 1.000 | **0.805** | **0.639** | 1.000 | 1.000 | **1.000** | **0.936** | 0.376 | 0.000 |  |  |  |  |  |  |  |  |  |  |  |  |
| 3 | PD | 0.000 | 0.000 | 0.000 | 1.000 | 0.000 | 0.051 | **0.243** | 1.000 | 0.000 | 0.000 | 0.000 | 0.008 | 1.000 | **1.000** |  |  |  |  |  |  |  |  |  |  |  |
| 3 | PDo | 0.000 | 0.000 | 0.000 | 1.000 | 0.000 | 0.000 | 0.149 | 1.000 | 0.000 | 0.000 | 0.000 | 0.000 | 1.000 | **1.000** | 0.000 |  |  |  |  |  |  |  |  |  |  |
| 3 | PS | 0.000 | 0.000 | 0.000 | 0.846 | 0.000 | 0.040 | **0.222** | 0.946 | 0.000 | 0.013 | 0.028 | 0.012 | 0.846 | **0.863** | 0.000 | 0.000 |  |  |  |  |  |  |  |  |  |
| 3 | PC | **1.000** | **0.768** | 1.000 | 1.000 | 1.000 | **0.897** | **0.775** | 1.000 | **1.000** | **1.000** | **0.957** | **0.587** | 0.000 | 0.000 | **1.000** | **1.000** | **0.937** |  |  |  |  |  |  |  |  |
| 4 | OB | 0.000 | 0.000 | 0.000 | 1.000 | 0.000 | 0.051 | **0.243** | 1.000 | 0.000 | 0.000 | 0.000 | 0.008 | 1.000 | **1.000** | 0.000 | 0.000 | 0.000 | **1.000** |  |  |  |  |  |  |  |
| 4 | SS | 0.000 | 0.000 | 0.000 | 1.000 | 0.000 | 0.012 | 0.200 | 1.000 | 0.000 | 0.000 | 0.000 | 0.000 | 1.000 | **1.000** | 0.000 | 0.000 | 0.000 | **1.000** | 0.000 |  |  |  |  |  |  |
| 5 | SO | 0.000 | 0.000 | 0.000 | 1.000 | 0.000 | 0.000 | 0.000 | 1.000 | 0.000 | 0.000 | 0.000 | 0.000 | 1.000 | 1.000 | 0.000 | 0.000 | 0.000 | 1.000 | 0.000 | 0.000 |  |  |  |  |  |
| 5 | VA | 0.000 | 0.000 | 0.000 | 1.000 | 0.000 | 0.000 | 0.000 | 1.000 | 0.000 | 0.000 | 0.000 | 0.000 | 1.000 | 1.000 | 0.000 | 0.000 | 0.000 | **1.000** | 0.000 | 0.000 | 0.000 |  |  |  |  |
| 5 | FI | 0.000 | 0.000 | 0.000 | 1.000 | 0.000 | 0.000 | 0.000 | 1.000 | 0.000 | 0.000 | 0.000 | 0.000 | 1.000 | 1.000 | 0.000 | 0.000 | 0.000 | **1.000** | 0.000 | 0.000 | 0.000 | 0.000 |  |  |  |
| 5 | WF | 0.000 | 0.000 | 0.000 | 1.000 | 0.000 | 0.000 | 0.000 | 1.000 | 0.000 | 0.000 | 0.000 | 0.000 | 1.000 | 1.000 | 0.000 | 0.000 | 0.000 | 1.000 | 0.000 | 0.000 | 0.000 | 0.000 | 0.000 |  |  |
| 6 | PG | 0.000 | 0.026 | 0.000 | **1.000** | 0.000 | 0.119 | **0.330** | 1.000 | 0.000 | 0.000 | 0.000 | **0.053** | 1.000 | **1.000** | 0.000 | 0.000 | 0.062 | **1.000** | 0.000 | 0.000 | 0.000 | 0.000 | 0.000 | 0.000 |  |
| 6 | CC | 0.000 | 0.000 | 0.000 | 1.000 | 0.000 | 0.071 | **0.269** | 1.000 | 0.000 | 0.000 | 0.000 | 0.023 | 1.000 | **1.000** | 0.000 | 0.000 | 0.019 | **1.000** | 0.000 | 0.000 | 0.000 | 0.000 | 0.000 | 0.000 | 0.000 |

*Abbreviations for sampling sites are given in Table S1. p < 0.05 in bold. Groupings 1: South China Sea; 2: Southern Philippines/East Java; 3: Malay Peninsula/West Java; 4: Gulf of Thailand; 5: Pacific Islands; 6: Guam.

**Table S3.** Pairwise Φ_ST_ among populations of *Sargassum polycystum* based on Rub spacer.

| Grp* | Site | BL | PP | HN | PA | IC | PR | TE | MA | KE | WJ | PK | PD | PDo | PS | PC | OB | VA | FI | PG |
| --- | --- | --- | --- | --- | --- | --- | --- | --- | --- | --- | --- | --- | --- | --- | --- | --- | --- | --- | --- | --- |
| 1 | BL |  |  |  |  |  |  |  |  |  |  |  |  |  |  |  |  |  |  |  |
| 1 | PP | 0.000 |  |  |  |  |  |  |  |  |  |  |  |  |  |  |  |  |  |  |
| 1 | HN | 0.000 | 0.000 |  |  |  |  |  |  |  |  |  |  |  |  |  |  |  |  |  |
| 1 | PA | 1.000 | 1.000 | 1.000 |  |  |  |  |  |  |  |  |  |  |  |  |  |  |  |  |
| 2 | IC | 0.000 | 0.000 | 0.000 | 1.000 |  |  |  |  |  |  |  |  |  |  |  |  |  |  |  |
| 2 | PR | 0.043 | 0.043 | 0.088 | 0.600 | 0.150 |  |  |  |  |  |  |  |  |  |  |  |  |  |  |
| 2 | TE | 0.000 | 0.000 | 0.000 | 1.000 | 0.000 | 0.000 |  |  |  |  |  |  |  |  |  |  |  |  |  |
| 2 | MA | 0.588 | 0.588 | 0.688 | 0.000 | 0.788 | 0.000 | 0.000 |  |  |  |  |  |  |  |  |  |  |  |  |
| 2 | KE | 0.000 | 0.000 | 0.000 | 0.833 | 0.017 | 0.000 | 0.000 | 0.346 |  |  |  |  |  |  |  |  |  |  |  |
| 3 | WJ | 0.000 | 0.000 | 0.000 | **1.000** | 0.000 | 0.218 | 0.000 | 0.857 | 0.054 |  |  |  |  |  |  |  |  |  |  |
| 3 | PK | 0.000 | 0.000 | 0.000 | 1.000 | 0.000 | 0.174 | 0.000 | 0.817 | 0.030 | 0.000 |  |  |  |  |  |  |  |  |  |
| 3 | PD | **0.418** | **0.418** | **0.476** | 0.651 | **0.558** | **0.274** | 0.000 | 0.266 | **0.374** | **0.640** | **0.590** |  |  |  |  |  |  |  |  |
| 3 | PDo | 0.331 | 0.331 | 0.417 | 0.700 | **0.532** | 0.086 | 0.000 | 0.147 | 0.188 | **0.636** | **0.573** | 0.000 |  |  |  |  |  |  |  |
| 3 | PS | **0.386** | **0.386** | **0.444** | 0.722 | **0.528** | **0.216** | 0.000 | 0.293 | **0.311** | **0.612** | **0.560** | 0.000 | 0.000 |  |  |  |  |  |  |
| 3 | PC | 0.000 | 0.000 | 0.000 | 1.000 | 0.000 | 0.166 | 0.000 | 0.809 | 0.026 | 0.000 | 0.000 | **0.580** | **0.560** | **0.550** |  |  |  |  |  |
| 4 | OB | 0.000 | 0.000 | 0.000 | 1.000 | 0.000 | 0.088 | 0.000 | 0.688 | 0.000 | 0.000 | 0.000 | **0.476** | 0.417 | **0.444** | 0.000 |  |  |  |  |
| 5 | VA | 0.000 | 0.000 | 0.000 | 1.000 | 0.000 | 0.000 | 0.000 | 0.000 | 0.000 | 0.000 | 0.000 | 0.207 | 0.000 | 0.167 | 0.000 | 0.000 |  |  |  |
| 5 | FI | 0.000 | 0.000 | 0.000 | 1.000 | 0.000 | 0.000 | 0.000 | 0.000 | 0.000 | 0.000 | 0.000 | 0.207 | 0.000 | 0.167 | 0.000 | 0.000 | 0.000 |  |  |
| 6 | PG | 0.000 | 0.000 | 0.000 | 1.000 | 0.000 | 0.131 | 0.000 | 0.763 | 0.006 | 0.000 | 0.000 | **0.534** | 0.499 | **0.504** | 0.000 | 0.000 | 0.000 | 0.000 |  |
| 6 | CC | 0.000 | 0.000 | 0.000 | 1.000 | 0.000 | 0.060 | 0.000 | 0.628 | 0.000 | 0.000 | 0.000 | **0.440** | 0.363 | **0.407** | 0.000 | 0.000 | 0.000 | 0.000 | 0.000 |

*Abbreviations for sampling sites are given in Table S1. p < 0.05 in bold. Groupings 1: South China Sea; 2: Southern Philippines/East Java; 3: Malay Peninsula/West Java; 4: Gulf of Thailand; 5: Pacific Islands; 6: Guam

**Table S4.** Pairwise Φ_ST_ among populations of *Sargassum polycystum* based on Cox3.

| Grp* | Site | BL | PP | HN | IC | TE | MA | KE | PK | PD | PC | OB | SS | MS | SO | VA | FI | PG | CC |
| --- | --- | --- | --- | --- | --- | --- | --- | --- | --- | --- | --- | --- | --- | --- | --- | --- | --- | --- | --- |
| 1 | BL |  |  |  |  |  |  |  |  |  |  |  |  |  |  |  |  |  |  |
| 1 | PP | 0.000 |  |  |  |  |  |  |  |  |  |  |  |  |  |  |  |  |  |
| 1 | HN | 0.000 | 0.000 |  |  |  |  |  |  |  |  |  |  |  |  |  |  |  |  |
| 2 | IC | 0.000 | 0.000 | 0.000 |  |  |  |  |  |  |  |  |  |  |  |  |  |  |  |
| 2 | TE | 1.000 | 1.000 | 1.000 | 1.000 |  |  |  |  |  |  |  |  |  |  |  |  |  |  |
| 2 | MA | 0.788 | 0.730 | 0.660 | 0.730 | 0.000 |  |  |  |  |  |  |  |  |  |  |  |  |  |
| 2 | KE | 0.017 | 0.000 | 0.000 | 0.000 | 0.625 | 0.346 |  |  |  |  |  |  |  |  |  |  |  |  |
| 2 | PK | 0.000 | 0.000 | 0.000 | 0.000 | 0.600 | 0.451 | 0.000 |  |  |  |  |  |  |  |  |  |  |  |
| 3 | PD | 0.000 | 0.000 | 0.000 | 0.000 | 1.000 | 0.711 | 0.000 | 0.000 |  |  |  |  |  |  |  |  |  |  |
| 3 | PC | 0.000 | 0.000 | 0.000 | 0.000 | 1.000 | 0.817 | 0.030 | 0.000 | 0.000 |  |  |  |  |  |  |  |  |  |
| 4 | OB | 0.000 | 0.000 | 0.000 | 0.000 | 1.000 | 0.711 | 0.000 | 0.000 | 0.000 | 0.000 |  |  |  |  |  |  |  |  |
| 4 | SS | 0.093 | 0.054 | 0.016 | 0.054 | 0.778 | 0.494 | 0.000 | 0.000 | 0.042 | 0.118 | 0.042 |  |  |  |  |  |  |  |
| 4 | MS | 0.000 | 0.000 | 0.000 | 0.000 | 1.000 | 0.000 | 0.000 | 0.000 | 0.000 | 0.000 | 0.000 | 0.000 |  |  |  |  |  |  |
| 5 | SO | 0.000 | 0.000 | 0.000 | 0.000 | 1.000 | 0.000 | 0.000 | 0.000 | 0.000 | 0.000 | 0.000 | 0.000 | 0.000 |  |  |  |  |  |
| 5 | VA | 0.000 | 0.000 | 0.000 | 0.000 | 1.000 | 0.000 | 0.000 | 0.000 | 0.000 | 0.000 | 0.000 | 0.000 | 0.000 | 0.000 |  |  |  |  |
| 5 | FI | 0.000 | 0.000 | 0.000 | 0.000 | 1.000 | 0.000 | 0.000 | 0.000 | 0.000 | 0.000 | 0.000 | 0.000 | 0.000 | 0.000 | 0.000 |  |  |  |
| 6 | PG | **1.000** | **1.000** | **1.000** | **1.000** | 0.000 | **0.935** | **0.868** | **0.811** | **1.000** | **1.000** | **1.000** | **0.947** | **1.000** | 1.000 | **1.000** | **1.000** |  |  |
| 6 | CC | **1.000** | **1.000** | **1.000** | **1.000** | 0.000 | **0.894** | **0.825** | **0.768** | **1.000** | **1.000** | **1.000** | **0.924** | **1.000** | 1.000 | **1.000** | **1.000** | 0.000 |  |

*Abbreviations for sampling sites are given in Table S1. p< 0.05 in bold. Groupings 1: South China Sea; 2: Southern Philippines/East Java; 3: Malay Peninsula/West Java; 4: Gulf of Thailand; 5: Pacific Islands; 6: Guam.

**Table S5.** Hierarchical Analysis of Molecular Variance (AMOVA) based on the three markers ITS2, Rub spacer and Cox3 from *Sargassum polycystum*.

| ITS2 | | | |
| --- | --- | --- | --- |
| Number of Groups | Groupings* | % var | Φ_CT_ |
| 6 | (BL, PP, HN, PA, NT)(IC, PR, TE, MA, KE)(WJ, PK, SE, MB, PD, PDo, PS, PC)(OB, SS)(SO, VA, FI, WF)(PG, CC) | -8.53 | -0.085 |
| 5 | (BL, PP, HN, PA, NT, MA, KE, SS, OB, IC)(TE, PR, PS, PD ,PDo,)(SE, PC, MB, WJ, PK)(SO, FI, WF, VA)(PG, CC) | 1.93 | 0.019 |
| 4 | (BL, HN, PA, NT, MA, KE, SS, OB)(TE, PR, PS, PD, PDo, SE, PC, MB, WJ, PK)(SO, FI, WF, VA)(PG, CC)(IC, PP) | -9.14 | -0.091 |
| Rub spacer | | | |
| Number of Groups | Groupings* | % var | Φ_CT_ |
| 5 | (BL, PP, HN, PA, OB, IC, PR, TE, PK, WJ)(PD, PDo, PS, PC)(CC, PG)(FI, VA)(MA, KE) | 9.40 | 0.094 |
| 4 | (BL, PP, HN, PA, OB, IC, PR, TE, PK, WJ, MA, KE)(PD, PDo, PS, PC)(PG, CC)(FI, VA) | 12.8 | 0.128 |
| Cox3 | | | |
| Number of Groups | Groupings* | % var | Φ_CT_ |
| 4 | (BL, HN, IC, MS, OB, PC, PD, PP, SS, TE, PK)(KE, MA)(PG, CC)(SO, VA, FI) | 82.80 | 0.828 |
| 3 | (BL, HN, IC, MS, OB, PC, PD, PP, SS, TE, PK, KE, MA)(CC, PG)(VA, FI, SO) | 85.38 | 0.854 |
| 4 | (BL, HN, IC, MS, OB, PC, PD, PP, SS, TE, KE, MA)(CC, PG)(VA, FI, SO)(PK) | 80.14 | 0.801 |

*Abbreviations for sampling sites are given in Table S1.

**Table S6.** Migration estimates of gene flow between subregions 1 to 6 and 95% confidence values as estimated by MIGRATE using ITS2.

| **Migration*** | **2.5%** | **25.0%** | **Mode** | **75.0%** | **97.5%** | **Median** | **Mean** |
| --- | --- | --- | --- | --- | --- | --- | --- |
| 2 to 1 | 0.0 | 0.0 | 0.3 | 402.7 | 834.7 | 487.0 | 493.0 |
| 3 to 1 | 68.7 | 528.7 | 963.0 | 986.7 | 993.3 | 541.0 | 529.1 |
| 4 to 1 | 126.0 | 662.7 | 976.3 | 994.7 | 1000.0 | 669.0 | 625.3 |
| 5 to 1 | 66.7 | 552.7 | 980.3 | 988.7 | 993.3 | 533.7 | 523.7 |
| 6 to 1 | 94.0 | 625.3 | 965.0 | 993.3 | 1000.0 | 632.3 | 594.7 |
| 1 to 2 | 0.0 | 0.0 | 21.7 | 453.3 | 927.3 | 453.7 | 469.3 |
| 3 to 2 | 0.0 | 0.0 | 0.3 | 446.0 | 936.0 | 446.3 | 464.6 |
| 4 to 2 | 0.0 | 0.0 | 51.0 | 455.3 | 923.3 | 475.0 | 484.0 |
| 5 to 2 | 0.0 | 0.0 | 0.3 | 440.7 | 898.0 | 461.0 | 474.1 |
| 6 to 2 | 59.3 | 508.0 | 980.3 | 986.7 | 990.7 | 520.3 | 514.3 |
| 1 to 3 | 0.0 | 0.0 | 0.3 | 413.3 | 839.3 | 477.0 | 485.8 |
| 2 to 3 | 131.3 | 631.3 | 981.0 | 993.3 | 1000.0 | 638.3 | 606.1 |
| 4 to 3 | 0.0 | 0.0 | 0.3 | 70.0 | 294.7 | 504.3 | 503.7 |
| 5 to 3 | 8.7 | 12.7 | 29.0 | 153.3 | 942.7 | 497.7 | 498.6 |
| 6 to 3 | 76.7 | 585.3 | 949.7 | 992.0 | 1000.0 | 593.7 | 565.2 |
| 1 to 4 | 0.0 | 0.0 | 0.3 | 455.3 | 938.7 | 459.7 | 474.2 |
| 2 to 4 | 0.0 | 0.0 | 0.3 | 435.3 | 935.3 | 441.7 | 461.9 |
| 3 to 4 | 0.0 | 0.0 | 21.0 | 416.0 | 926.0 | 416.3 | 444.4 |
| 5 to 4 | 0.0 | 0.0 | 13.7 | 418.7 | 888.0 | 464.3 | 477.2 |
| 6 to 4 | 66.0 | 568.0 | 980.3 | 992.7 | 998.0 | 575.7 | 551.6 |
| 1 to 5 | 0.0 | 0.0 | 0.3 | 398.0 | 778.7 | 489.0 | 493.5 |
| 2 to 5 | 52.7 | 530.7 | 980.3 | 989.3 | 993.3 | 540.3 | 528.0 |
| 3 to 5 | 0.0 | 0.0 | 14.3 | 435.3 | 655.3 | 485.7 | 491.5 |
| 4 to 5 | 77.3 | 570.0 | 981.0 | 993.3 | 998.7 | 577.0 | 552.4 |
| 6 to 5 | 86.0 | 616.7 | 980.3 | 994.7 | 1000.0 | 623.0 | 586.3 |
| 1 to 6 | 0.0 | 0.0 | 10.3 | 428.0 | 931.3 | 428.3 | 452.2 |
| 2 to 6 | 0.0 | 0.0 | 17.7 | 462.7 | 928.0 | 463.0 | 475.9 |
| 3 to 6 | 0.0 | 0.0 | 12.3 | 424.0 | 929.3 | 424.3 | 449.7 |
| 4 to 6 | 0.0 | 0.0 | 35.7 | 473.3 | 816.7 | 483.7 | 490.2 |
| 5 to 6 | 0.0 | 0.0 | 31.0 | 443.3 | 935.3 | 443.7 | 463.6 |

*Subregions 1: South China Sea and Gulf of Thailand; 2: West coast of Malay Peninsula; 3: West Java; 4: Celebes Sea and Flores Sea; 5: Guam; 6: Pacific Islands.

**Table S7.** Migration estimates of gene flow between subregions 1 to 6 and 95% confidence values as estimated by MIGRATE using Rub spacer.

| **Migration*** | **2.5%** | **25.0%** | **Mode** | **75.0%** | **97.5%** | **Median** | **Mean** |
| --- | --- | --- | --- | --- | --- | --- | --- |
| 2 to 1 | 92.0 | 537.3 | 979.7 | 990.7 | 994.0 | 545.7 | 531.2 |
| 3 to 1 | 64.7 | 562.7 | 980.3 | 992.7 | 998.0 | 570.3 | 548.5 |
| 4 to 1 | 57.3 | 568.0 | 947.7 | 990.7 | 996.0 | 554.3 | 537.7 |
| 5 to 1 | 63.3 | 552.7 | 980.3 | 992.0 | 996.0 | 549.0 | 533.4 |
| 6 to 1 | 76.0 | 589.3 | 975.7 | 993.3 | 1000.0 | 597.0 | 566.4 |
| 1 to 2 | 0.0 | 0.0 | 0.3 | 442.0 | 936.7 | 446.3 | 465.3 |
| 3 to 2 | 0.0 | 0.0 | 23.0 | 381.3 | 876.0 | 473.0 | 482.6 |
| 4 to 2 | 0.0 | 0.0 | 0.3 | 472.0 | 905.3 | 475.0 | 483.6 |
| 5 to 2 | 0.0 | 0.0 | 0.3 | 380.0 | 656.7 | 485.0 | 491.0 |
| 6 to 2 | 281.3 | 527.3 | 980.3 | 990.7 | 992.7 | 533.7 | 522.7 |
| 1 to 3 | 0.0 | 0.0 | 0.3 | 416.0 | 630.7 | 489.7 | 493.7 |
| 2 to 3 | 492.7 | 656.0 | 952.3 | 983.3 | 984.7 | 506.3 | 505.8 |
| 4 to 3 | 143.3 | 564.7 | 980.3 | 990.7 | 993.3 | 536.3 | 525.2 |
| 5 to 3 | 261.3 | 562.7 | 980.3 | 990.7 | 992.0 | 528.3 | 519.5 |
| 6 to 3 | 68.0 | 588.7 | 980.3 | 994.7 | 1000.0 | 595.0 | 563.8 |
| 1 to 4 | 0.0 | 0.0 | 0.3 | 352.0 | 690.0 | 488.3 | 493.3 |
| 2 to 4 | 369.3 | 542.0 | 980.3 | 989.3 | 990.7 | 521.0 | 514.4 |
| 3 to 4 | 74.7 | 551.3 | 980.3 | 991.3 | 996.7 | 560.3 | 541.3 |
| 5 to 4 | 256.7 | 554.7 | 965.7 | 990.7 | 992.0 | 535.7 | 524.5 |
| 6 to 4 | 80.7 | 608.7 | 980.3 | 996.0 | 1000.0 | 614.3 | 576.8 |
| 1 to 5 | 0.0 | 0.0 | 51.7 | 468.0 | 861.3 | 477.0 | 485.2 |
| 2 to 5 | 58.7 | 520.7 | 980.3 | 989.3 | 991.3 | 523.7 | 516.2 |
| 3 to 5 | 307.3 | 555.3 | 954.3 | 986.7 | 988.0 | 518.3 | 513.0 |
| 4 to 5 | 317.3 | 574.7 | 980.3 | 987.3 | 988.0 | 510.3 | 507.4 |
| 6 to 5 | 71.3 | 569.3 | 980.3 | 992.7 | 998.0 | 577.0 | 552.7 |
| 1 to 6 | 0.0 | 0.0 | 0.3 | 440.0 | 918.0 | 440.3 | 461.0 |
| 2 to 6 | 0.0 | 0.0 | 2.3 | 442.0 | 590.7 | 483.0 | 489.6 |
| 3 to 6 | 0.0 | 0.0 | 19.0 | 435.3 | 624.7 | 481.7 | 488.2 |
| 4 to 6 | 0.0 | 0.0 | 0.3 | 400.7 | 692.0 | 482.3 | 488.6 |
| 5 to 6 | 0.0 | 0.0 | 0.3 | 290.7 | 489.3 | 489.7 | 494.5 |

*Subregions 1: South China Sea and Gulf of Thailand; 2: West coast of Malay Peninsula; 3: West Java; 4: Celebes Sea and Flores Sea; 5: Guam; 6: Pacific Islands.

**Table S8.** Migration estimates of gene flow between subregions 1 to 6 and 95% confidence values as estimated by MIGRATE using Cox3.

| **Migration*** | **2.5%** | **25.0%** | **Mode** | **75.0%** | **97.5%** | **Median** | **Mean** |
| --- | --- | --- | --- | --- | --- | --- | --- |
| 2 to 1 | 129.3 | 706.7 | 980.3 | 997.3 | 1000.0 | 710.3 | 651.7 |
| 3 to 1 | 0.0 | 0.0 | 0.3 | 315.3 | 400.0 | 497.7 | 498.9 |
| 4 to 1 | 128.0 | 591.3 | 973.7 | 994.7 | 1000.0 | 597.7 | 565.6 |
| 5 to 1 | 268.7 | 542.0 | 971.7 | 992.7 | 994.7 | 549.7 | 533.3 |
| 6 to 1 | 605.3 | 749.3 | 980.3 | 986.7 | 988.7 | 498.3 | 500.0 |
| 1 to 2 | 0.0 | 0.0 | 0.3 | 427.3 | 931.3 | 427.7 | 452.2 |
| 3 to 2 | 0.0 | 0.0 | 0.3 | 413.3 | 916.7 | 413.7 | 442.0 |
| 4 to 2 | 0.0 | 0.0 | 0.3 | 460.0 | 894.7 | 460.3 | 474.5 |
| 5 to 2 | 0.0 | 0.0 | 0.3 | 438.7 | 918.7 | 439.0 | 459.8 |
| 6 to 2 | 0.0 | 0.0 | 1.7 | 418.0 | 898.0 | 418.3 | 445.8 |
| 1 to 3 | 110.7 | 542.0 | 961.0 | 990.0 | 994.7 | 551.7 | 535.7 |
| 2 to 3 | 149.3 | 714.7 | 969.0 | 996.7 | 1000.0 | 719.7 | 663.7 |
| 4 to 3 | 80.0 | 614.0 | 965.0 | 994.0 | 1000.0 | 621.0 | 583.9 |
| 5 to 3 | 75.3 | 560.0 | 980.3 | 993.3 | 997.3 | 567.7 | 545.5 |
| 6 to 3 | 108.7 | 514.0 | 980.3 | 990.0 | 991.3 | 523.7 | 516.4 |
| 1 to 4 | 0.0 | 0.0 | 0.3 | 57.3 | 278.0 | 506.3 | 505.2 |
| 2 to 4 | 90.7 | 634.0 | 980.3 | 996.0 | 1000.0 | 639.0 | 596.4 |
| 3 to 4 | 4.0 | 9.3 | 21.7 | 435.3 | 804.7 | 492.3 | 495.0 |
| 5 to 4 | 184.0 | 544.0 | 980.3 | 990.0 | 991.3 | 525.0 | 517.6 |
| 6 to 4 | 0.0 | 0.0 | 54.3 | 192.7 | 376.7 | 497.7 | 499.8 |
| 1 to 5 | 434.0 | 672.0 | 960.3 | 984.0 | 985.3 | 506.3 | 505.0 |
| 2 to 5 | 92.7 | 645.3 | 980.3 | 996.7 | 1000.0 | 649.7 | 605.0 |
| 3 to 5 | 0.0 | 0.0 | 17.0 | 401.3 | 764.7 | 485.7 | 490.6 |
| 4 to 5 | 66.7 | 546.0 | 980.3 | 992.0 | 996.0 | 554.3 | 537.2 |
| 6 to 5 | 0.0 | 0.0 | 36.3 | 274.0 | 547.3 | 491.7 | 495.6 |
| 1 to 6 | 131.3 | 575.3 | 980.3 | 989.3 | 990.7 | 519.7 | 513.6 |
| 2 to 6 | 86.0 | 620.7 | 965.7 | 994.0 | 1000.0 | 627.0 | 589.4 |
| 3 to 6 | 486.0 | 734.7 | 933.0 | 983.3 | 984.7 | 503.0 | 503.0 |
| 4 to 6 | 62.7 | 543.3 | 959.7 | 990.7 | 995.3 | 552.3 | 536.2 |
| 5 to 6 | 127.3 | 514.7 | 954.3 | 987.3 | 990.0 | 526.3 | 518.5 |

*Subregions 1: South China Sea and Gulf of Thailand; 2: West coast of Malay Peninsula; 3: West Java; 4: Celebes Sea and Flores Sea; 5: Guam; 6: Pacific Islands.
