# Supplementary figures and images for: Homogeneous Population of the Brown Alga Sargassum polycystum in Southeast Asia: Possible Role of Recent Expansion and Asexual Propagation
Source: PLoS One. 2013 Oct 17;8(10):e77662. doi: 10.1371/journal.pone.0077662 (PMC3798308; doi:10.1371/journal.pone.0077662)

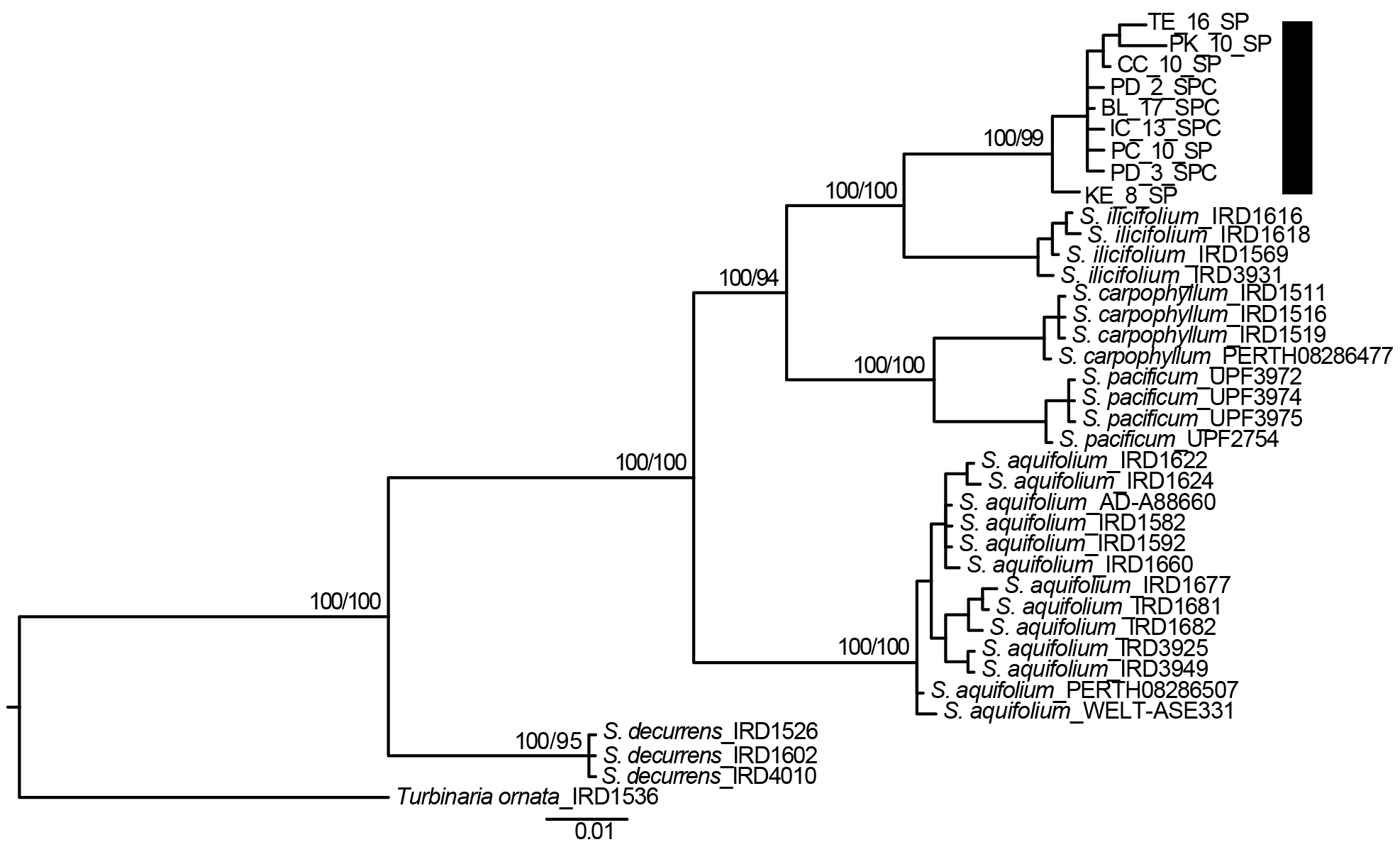

Supplement: Figure S1 — Phylogenetic tree of representative sequences from each haplotype of combined ITS2, Rub spacer and Cox3 sequences from Sargassumpolycystum with other Sargassum spp. in subgenus Sargassum. Voucher numbers of sequences from Genbank are presented after species name. Representative sequences from each haplotype of S. polycystum are labeled in black bar. Turbinaria ornata is used as outgroup. Posterior probabilities of Bayesian Inference and boostrap value of Maximum Likelihood are shown. Tables of sample localities, diversity indices, neutrality tests; Pairwise ST; Hierarchical Analysis of Molecular Variance (AMOVA) and Migration estimates of gene flow between subregions of three genes of S. polycystum. (TIF) [file pone.0077662.s002.tif]

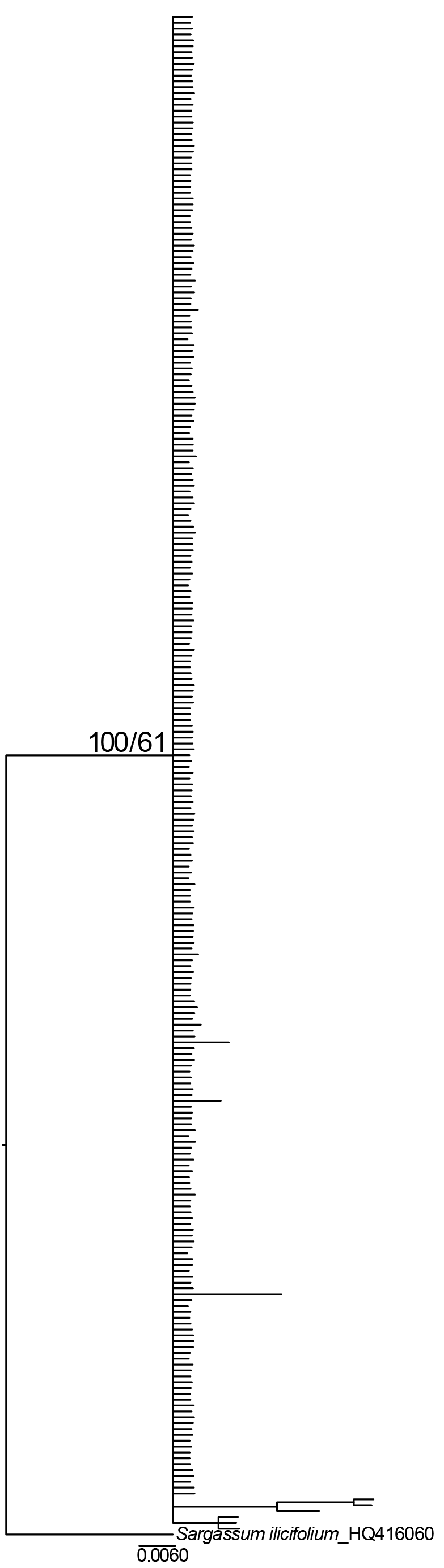

Supplement: Figure S2 — Phylogenetic tree inferred from ITS2 using Sargassumilicifolium as outgroup. Posterior probabilities of Bayesian Inference and boostrap values of Maximum likelihood are shown. (TIF) [file pone.0077662.s003.tif]

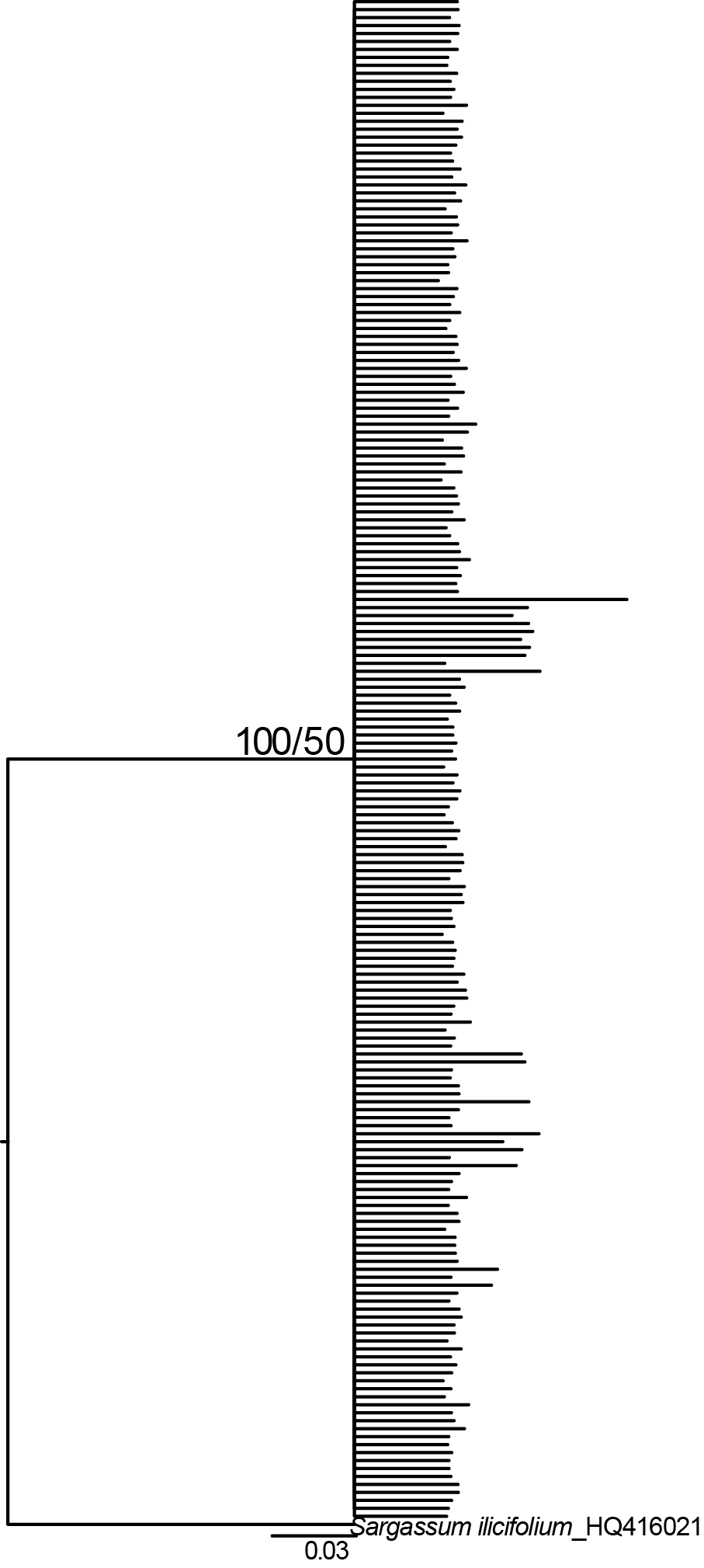

Supplement: Figure S3 — Phylogenetic tree inferred from Rub spacer using Sargassumilicifolium as outgroup. Posterior probabilities of Bayesian Inference and boostrap values of Maximum likelihood are shown. (TIF) [file pone.0077662.s004.tif]

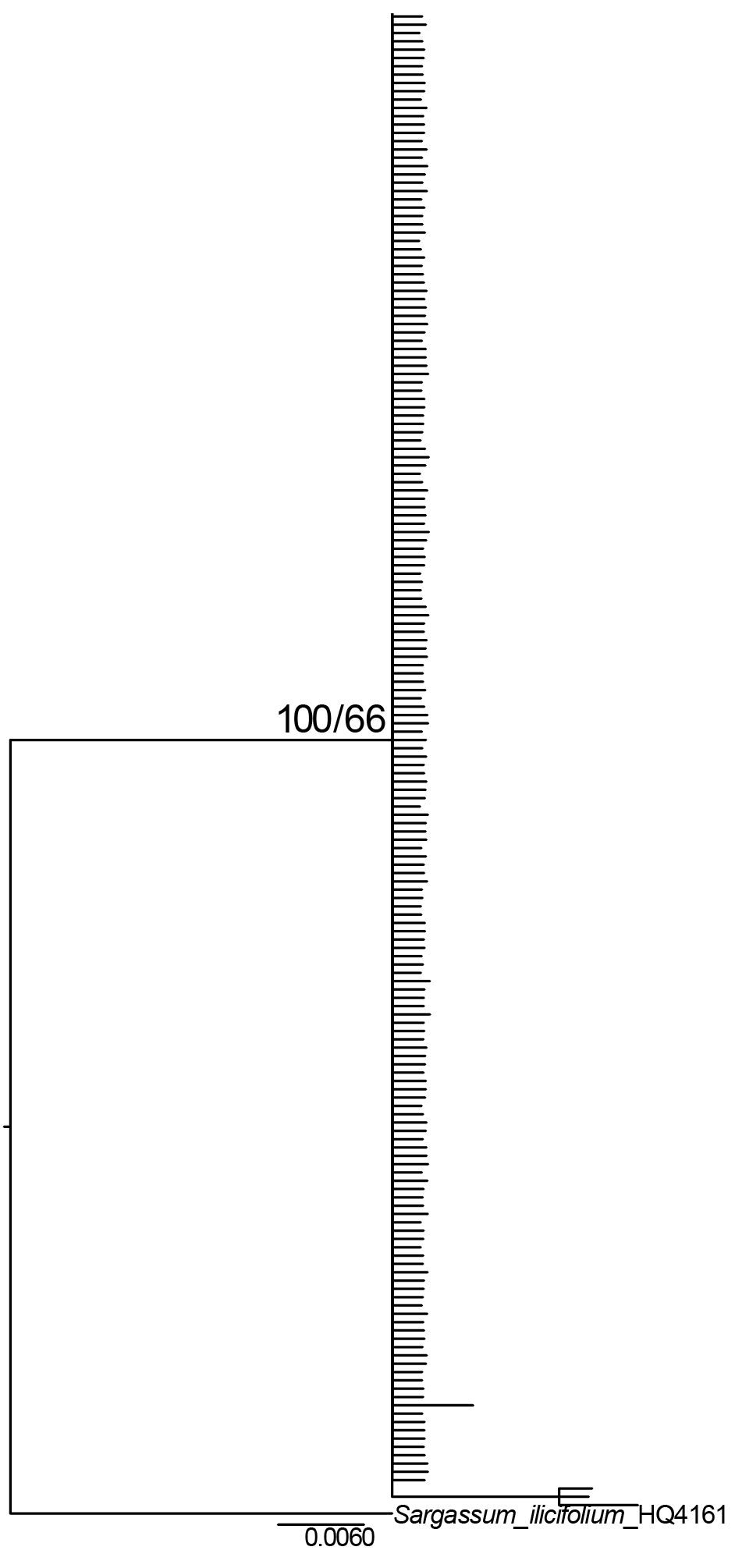

Supplement: Figure S4 — Phylogenetic tree inferred from Cox3 using Sargassumilicifolium as outgroup. Posterior probabilities of Bayesian Inference and boostrap values of Maximum likelihood are shown. (TIF) [file pone.0077662.s005.tif]

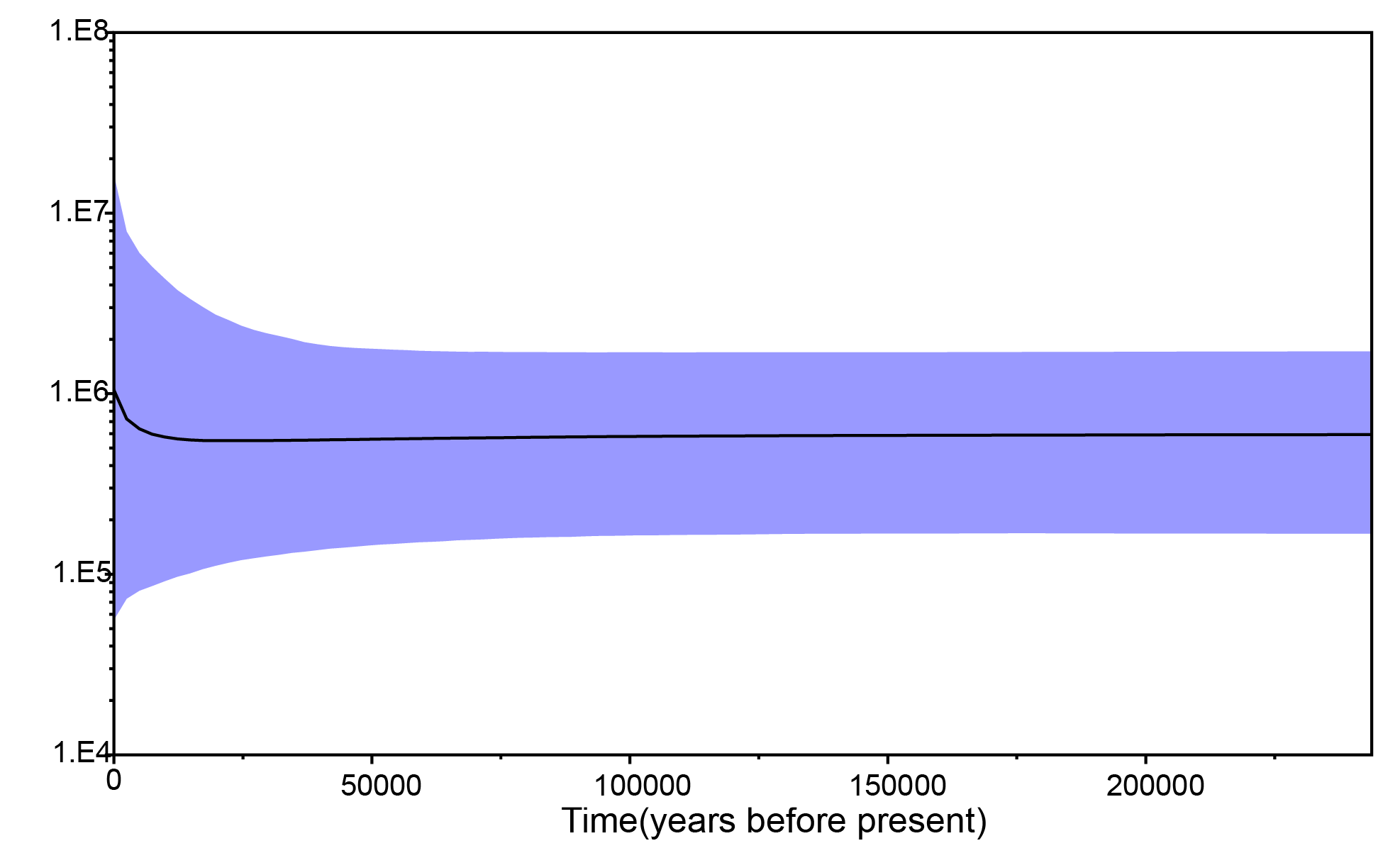

Supplement: Figure S5 — Bayesian Skyline Plot of Cox3 in effective population size with function of time (year before present). 95% confidence interval is shown in blue. (TIF) [file pone.0077662.s006.tif]
